# The Call for Nursing Reform

**Published:** 1907-04-06

**Authors:** 


### Nursing Administration.

### THE CALL FOR NURSING REFORM.

Few people will be found to contend that all is as it should be in the training and supply of nurses to the public in the present day. The evils incessantly complained of differ widely in character, and, as is generally the case, those most loudly talked about are of relatively small importance compared with those which are known only to the initiated. The defects of the existing system fall under three headings. There are imperfections in the training of the trained nurse; there is no machinery for dealing with misconduct and deterioration in the trained nurse; there is no well defined line of demarcation which the public can easily recognise between the trained nurse and the untrained attendant. Now, of these three incomparably the most serious is that which relates to the making of the nurse, and the really vital question for nursing reform is the introduction of system into their course of training. It is undoubtedly true that the education of nurses is making good progress all along the line, and that every year marks the establishment of a higher standard in successive training schools; but progress in this direction remains where it originally started, in the hands of individuals, and depends to a regrettable extent on the measure of time and zeal expended on it by those concerned in the work. It is in the power of a lax matron to minimise the advantages secured by her predecessor for probationers, by want of justice in distributing the work, and by rendering attendance at lectures and preparation for examinations purely perfunctory. The nurses of such a matron will continue to receive their certificates automatically at the conclusion of their term of service, and there will be no open indication that the standard of the training school has gone down. Or, again, a zealous matron may be hampered at every turn by the provisions of an antiquated system which she is powerless to alter. She may be keenly aware that her probationers do not receive the advantages which, in her opinion, are their due, but she has no alternative but to sign their certificates after doing the best for them in her power. The great argument against the present system of *laissez faire* is that it is precisely those who have done most to advance the education of their nurses who perceive most clearly the need for decisive reform in the curriculum. But in view of Miss Amy Hughes' recent statement that nurses frequently present themselves to her provided with a three-year certificate from reputable hospitals who yet prove to be incapable of either giving an ordinary douche or passing the catheter, it can hardly be said that this is a matter which concerns alone those who hold advanced views.

Again, amidst the large number of nurses who complete their training year by year, it is hardly conceivable that there should not be some who prove themselves to be unworthy of trust. But in the existing state of affairs there is no means by which the nurse's certificate can be confiscated and her entry into private households on confidential terms, even after the grossest misconduct, be prohibited. The heads of training schools cannot add to their other duties that of acting amateur detective towards former members of the staff, and once certificated always certificated is the common practice. Disciplinary powers for dealing with unsatisfactory members of this large body of women are urgently needed in the interests of both nurses and the public.

Lastly, there is the question of crisply defining the term "trained nurse." In our opinion the much lamented competition by untrained women is likely to die quietly away as the knowledge of what training involves becomes more widely diffused. Every day it is becoming more difficult for a woman whose training is either defective or unusual in character to get employment as a private nurse. For the public rarely and for the most part solely for chronic cases, comes into direct contact with the nurse without the intermediary of doctor or nursing agency, and hence the opportunity of trading on general ignorance as to the qualification is cut away. The nurse who gets her own cases is becoming extinct. Doctors seldom now care to preserve the addresses of private nurses only to find in an emergency that they are engaged elsewhere, and thus the nurse who is engaged for private work has to satisfy not only the requirements of the nursing agency to which she is attached, but also the requirements of the doctor who supports the agency, and is prompt to say, "Don't send me that nurse again," if he finds she does not understand her business. Private venture nursing agencies of a wholly unsatisfactory character continue to exist and will continue until placed under official inspection, but it does not in the present day pay any reputable agency to staff inadequately-trained nurses. Grave inconvenience does undoubtedly arise from having no definite standard of knowledge and experience which shall differentiate the "trained nurse" from the women "who have learned a little nursing," but it is the imperfectly constituted training schools which perpetuate the evil. Hospitals of every size, from the London with its 929 beds, to the cottage hospital with six, undertake to train probationers and issue certificates which form the passport to employment. Somewhere in the descending scale lies the point at which the line ought to be drawn, prescribing what measure of experience can suffice to produce the nurse. The course of training which entitles to a certificate runs down in similar fashion from the most advanced curriculum set by experienced lecturers, in which probationers enthusiastically compete for distinction, to a smattering of fourth-standard physiology, backed by prosy and time-honoured dissertations on the art of nursing. Will anyone dispute that a standard of knowledge should be set below which the performance of nursing duties ought not to be counted as "training" in any true sense of the word? It is not the pseudo nurse who constitutes the most serious cause for disquiet in the nursing world to-day. She is often merely a very worthy woman who has entered her calling by the wrong door. It is the training of the trained nurse which is calling for reform. It is want of system in the curriculum, want of any security for the proper carrying out of what system exists, and want of control over trained nurses and nursing agencies, which are responsible for the evils all superintendents of nursing have reason to deplore.

